# Antibiotic resistance patterns and extended-spectrum β-lactamase production among *Acinetobacter *spp. isolated from an intensive care Unit of a hospital in Kerman, Iran

**DOI:** 10.1186/2047-2994-1-1

**Published:** 2012-01-26

**Authors:** Mohammad Reza Shakibaie, Saied Adeli, Mohammad Hosain Salehi

**Affiliations:** 1 Department of Microbiology, Kerman University of Medical Sciences, Kerman, Iran

**Keywords:** *Acinetobacter *spp, antibiotic resistance, MIC, extended-spectrum β-lactamase

## Abstract

**Background:**

The global increase in multidrug resistance of *Acinetobacter *spp. has created widespread problems in the treatment of patients in intensive care units (ICUs) of hospitals. To assess the sensitivity of *Acinetobacter *isolates to antibiotics routinely used in ICUs, we investigated antibiotic resistance patterns and extended-spectrum β-lactamase (ESBL) production among *Acinetobacter *spp. isolated from the ICU of a university hospital in Kerman, Iran.

**Methods:**

Fifteen isolates of *Acinetobacter *spp. were recovered from one hundred clinical specimens collected from the ICU of Afzalipoor Hospital in Kerman, Iran, from October 2010 to June 2011. Preliminary antibiotic sensitivity testing was carried out using the disk-diffusion breakpoint assay, and MICs of different antibiotics were determined using the E-test. ESBL production was detected by a double-disk synergy test and confirmed by a phenotypic confirmatory test. Substrate hydrolysis in the presence and absence of the following inhibitors was carried out using the rapid fixed-time method: para-chloromercuribenzoate (p-CMB), clavulanic acid, sulbactam, and NaCl.

**Results:**

Overall, 73.3% of the isolates were resistant to imipenem (MIC range 240-128 µg/mL) and 66% to ciprofloxacin (MIC range 240-64 ± 0.08 µg/mL). All of the isolates were fully resistant (MIC 240 µg/mL) to piperacillin, while 93.3%, 53.3%, and 93.3% were resistant to piperacillin + tazobactam (MIC 240 µg/mL), amikacin (MIC range 128-16 µg/mL), and cefepime (MIC range 240-60 µg/mL), respectively. The isolates were also resistant to chloramphenicol and tetracycline: MICs of these two agents were **≥ **240 µg/mL. The test for ESBL production was positive for only three isolates (nos. 1, 10, and 15). The rate of substrate hydrolysis was highest in the presence of p-CMB (80.2 **± **0.02) and lowest in the presence of NaCl (2.1 **± **0.01) (*P *≤ 0.05).

**Conclusions:**

Many isolates of *Acinetobacter *spp. are resistant to almost all antibiotics routinely used in the ICU of our hospital, including imipenem, ciprofloxacin, and piperacillin + tazobactam. Three isolates were ESBL producers. The other isolates exhibited high resistance to β-lactams, but they did not produce any ESBL enzymes.

## Introduction

*Acinetobacter *is a genus of gram-negative bacteria belonging to the Gammaproteobacteria. They are nonmotile, oxidase negative, highly pleomorphic and usually occur in pairs. The genus *Acinetobacter *has occupied an increasingly important position as an opportunistic pathogen in the hospital environment. The contribution of *Acinetobacter *spp. to nosocomial infection has increased over the past three decades, and many outbreaks of hospital infection involving *Acinetobacter *spp. have been reported worldwide [1-3].

Although generally regarded as commensals of human skin and the human respiratory tract, *Acinetobacter *spp. have also been implicated as the cause of serious infectious diseases such as pneumonia, urinary tract infections, endocarditis, wound infections, meningitis, and septicemia, involving mostly patients with impaired host defenses [2]. *Acinetobacter *spp. have emerged as particularly important organisms late-onset ventilator associated pneumonia in the in tensive care unit (ICU). This is probably related, at least in part, to the increasingly invasive diagnostic and therapeutic procedures used in hospital ICUs in recent years [4-6].

*Acinetobacter *spp. have acquired resistance to almost all currently available antimicrobial agents, including the aminoglycosides, the quinolones, and broad-spectrum β-lactams. The spectrum of antibiotic resistance of these organisms, together with their survival capabilities, makes them a threat in hospital environments, as documented by recurring outbreaks both in highly developed countries and elsewhere. Most strains are resistant to cephalosporins, while resistance to carbapenems is being reported increasingly [7,8]. One particular attribute of these strains is the production of extended-spectrum beta-lactamase (ESBL) enzymes that confer resistance to β-lactams [9]. Guillou et al. [10] screened 100 isolates of *Acinetobacter *spp. and found that 81% of the strains produced two types of β-lactamases (TEM and CARB).

*Acinetobacter baumannii *hospital isolates produce mainly cephalosporinase-type enzyme and are inhibited by 25 mM of clavulanic acid but not by 1 mM EDTA or 100 mM para-chloromercuribenzoate (p-CMB). The β-lactamases produced by *Acinetobacter lwoffii *ULA-501, *A. baumannii *ULA-187, and *A. baumannii *AC-14 strains have been purified and characterized, and their kinetic interactions with several β-lactam molecules, including substrates and inhibitors, have been studied in detail [11]. Three β-lactamase enzymes were identified and appeared to be cephalosporinase-type β-lactamases with different acylation efficiencies (kcat/Km ratio values). Their hydrolytic activities were inhibited by benzylpenicillin, piperacillin, and cefotaxime, none of which behaved as substrates for the enzyme. Carbenicillin was a substrate for the β-lactamase from *A. lwoffii *ULA-501, although it acted as a transient inactivator of the enzymes produced by the two *A. baumannii *strains. Clavulanic acid was unable to inactivate the three β-lactamases, whereas sulbactam behaved as an inactivator only at a high concentration (1 mM) that was difficult to achieve during antibiotic therapy [11].

Kim et al. [7] studied the prevalence and diversity of carbapenemases among imipenem-nonsusceptible *Acinetobacter *isolates in Korea. A total of 190 imipenem-nonsusceptible *Acinetobacter *isolates from 12 Korean hospitals in 2007 were used to determine species, prevalence, and antimicrobial susceptibility of OXA carbapenemase-producing and metallo-β-lactamase-producing isolates. *bla*_OXA-23 _-like and ISAba1-associated *bla*_OXA-51 _-like genes were detected in 80% and 12% of 178 imipenem-nonsusceptible *Acinetobacter *baumannii isolates, respectively.

Sinha et al. [9] recovered 150 clinical isolates of *Acinetobacter *and identified them using various phenotypic tests. Antibiotic susceptibility was determined by the standard disk-diffusion method. Most isolates were resistant to the antibiotics tested, including the third-generation cephalosporins. ESBL production was detected in 28% of the isolates. In the double-disk approximation test, most of the ESBLs in *Acinetobacter *isolates could be detected with cefepime and cefotaxime.

In one study in the UK, the antimicrobial susceptibility of *Acinetobacter *obtained from clinical specimens in 54 laboratories was investigated. The majority of the isolates were found to be more resistant to cefotaxime, ceftazidime, piperacillin, piperacillin + tazobactam, gentamicin, and tetracycline than the other gram negative bacteria [12].

Little information is available on the antibiotic resistance of *Acinetobacter *spp. isolated from hospitals in Iran. Khosroshahi and Sharifi [13] recovered 400 isolates from ICU patients in four university hospitals in Isfahan, Iran, of which 15 (3.75%) belonged to *A. baumannii*. Antibiotic sensitivity testing showed four (26.6%) isolates were resistant to imipenem and meropenem. Similarly, Farhani et al. [14] recovered 60 isolates of *Acinetobacter *spp. from Shahid Beheshti Hospital in Kashan, Iran. Among these, 48 were *A. baumannii*, six were *A. lwoffi*, and six were other *Acinetobacter *spp. They were resistant to amikacin, tobramycin, ampicillin + sulbactam, and imipenem.

To assess the level of sensitivity to antibiotics routinely used in the ICU of our hospital and to determine the production of ESBLs, we investigated the antibiotic resistance pattern and ESBL production in *Acinetobacter *spp. isolated from the ICU of the Afzalipoor Hospital in Kerman, Iran.

## Methods

### Source of bacteria

More than one hundred clinical specimens were collected from the ICU of Afzalipoor Hospital (the main university hospital) in the city of Kerman, Iran, from October 2010 to June 2011. The majority of the patients were hospitalized for 4 days, and 73% of them were on ventilator-assisted life support. The average age of the patients was 63 **± **0.8 years. Specimens of lung aspirates, blood, or urine were collected by a laboratory technician and transferred immediately to the Microbiology Department of the Kerman University of Medical Sciences in sterile screw-cap tubes containing 5 mL of tryptic soy broth (TSB) medium. Prior to collection of the specimens, criteria such as previous antimicrobial therapy, immunosuppression, and presence of bacteremia due to other pathogens before and after colonization by *Acinetobacter *were taken into consideration.

### Bacterial identification

Isolates were identified preliminarily by the chromosomal transformation assay [15] using an auxothrophic strain of *Acinetobacter calcoaceticus *BD143 trpE27, and species identification was carried out using the biochemical and sugar utilization tests as described by Bouvet and Grimont [16]. Isolates were further identified by Gram stain, motility, characteristics on nutrient agar and Cysteine-Lactose-Electrolyte-Deficient (CLED) agar, catalase and oxidase tests, acidity or alkalinity in triple sugar iron (TSI) agar slants, growth on citrate agar slants, hemolytic patterns on blood agar, glucose oxidation in Hugh and Leifson medium containing 1% glucose, and ability to grow at 44°C.

### Antibiotic susceptibility tests

Antibiotic sensitivity of the isolates was determined using the Kirby-Bauer disk-diffusion breakpoint assay on Mueller-Hinton agar using Oxoid disks (purchased from Hi-Media, India) as recommended previously by the Clinical and Laboratory Standards Institute (CLSI, previously called NCCLS) (2007 guidelines) [17]. MICs of different antibiotics were determined using the E-test (Hi-Media). Susceptibility to the following antimicrobial agents was tested: cefotaxime, cefepime, cefazolin, ciprofloxacin, piperacillin, piperacillin + tazobactam, ceftazidime, imipenem, tetracycline, gentamicin, amikacin, and chloramphenicol. Isolates were considered susceptible if the MIC was **≤ **2 µg/mL and resistant if the MIC was **≥ **8 µg/mL. A standard culture of *A. calcoaceticus *BD413 was used as sensitive bacterium at an inoculum of 1.5 × 10^7^CFU/mL.

### Detection of ESBL

Two disk-diffusion methods were employed in this investigation, both described previously [18]. Briefly, the first method is based on the original double-disk standard test (DDST); isolates are examined for the expansion of the cefotaxime + clavulanic acid inhibition zone adjacent to disks containing cefotaxime alone and amoxicillin + clavulanic acid 30 + 10 mg. In the second method, a disk containing 30 mg of ceftazidime is placed adjacent to a combination disk containing ceftazidime (30 mg) with clavulanic acid (10 mg) on sterile Mueller-Hinton agar inoculated with ESBL-positive and non-ESBL-producing standard cultures of *Acinetobacter *isolates. An expansion of > 5 mm or 50% (according to the manufacturer's guidelines) indicates ESBL production. The antibiotic disks described above were obtained from Oxoid, Mast, and Beckton Dickinson, UK.

### Substrate hydrolysis in the presence and absence of inhibitors

In order to eliminate the possibility of false-positive ESBL tests due to intrinsic susceptibility of is olates to β-lactamase inhibitors (which could result in false-positive or false-negative tests), the rapid fixed-time assay was used to measure β-lactamase activity, based on the reduction of iodine by hydrolysis of cefotaxime/ceftazidime under an ultraviolet light spectrophotometer at 450 nm. In this case, the cultures were centrifuged at 8,000 rpm at 4°C for 15 minutes after overnight growth in Luria-Bertani broth medium, and the cell pellet was washed with 0.01 M sterile phosphate buffer (pH 8.0). The suspension was sonicated with a Lab sonic sonicator (Germany) for 15 seconds using a 50% on/off pulsed cycle. Sonication was followed by freeze-thawing at -70°C for 10 minutes. The sonicated cells were centrifuged at 12,000 rpm for 10 minutes and then observed microscopically to see the disintegrated bacterial cells.

The sonicated solutions of the *Acinetobacter *isolates were then diluted with 2.5 mL of 0.01 M phosphate buffer (pH 8.0). To this preparation, 0.5 mL of 200 µg/mL substrate (cefotaxime and ceftazidime) was added separately and incubated at room temperature for 30 minutes. In case of β-lactamase inhibitors, 0.5 mL of 0.5 mM p-CMB (Fluka, Germany), 100 mM NaCl, 200 µg/mL sulbactam/clavulanic acid, and 200 µg/mL cloxacillin were added to the crude enzyme preparation 15 minutes before addition of the substrates. The reaction was stopped by the addition of 5 mL iodine reagent containing 0.32 N I_2 _and 1.2 M KI with rapid stirring at room temperature. Absorbance was measured at 540 nm, and the results were compared with preparation containing the inhibitors. Simultaneously, two blanks, one containing 3 mL phosphate buffer (pH 7.5) and 5 mL of iodine reagent and the other containing 5 mL of phosphate buffer (pH 7.5), were run alongside of the tests.

### Statistical analysis

All analyses were performed using SPSS, version 16.0 (SPSS Inc, Chicago, IL, USA). All *P *values were two-tailed; *P ***≤ **0.05 was considered statistically significant. Means and standard deviations (SD) were calculated as required for numerical variables.

## Results

### Bacterial isolates

From October 2010 to June 2011, more than one hundred specimens were collected from the ICU of Afzalipoor Hospital in Kerman, Iran. From these specimens, fifteen isolates were iden tified as *Acinetobacter *spp. by different biochemical tests and a chromosomal transformation assay using an auxothrophic strain of *A. calcoaceticus *BD413trpE27. The study was conducted in the ICU of Afzalipoor Hospital in Kerman, Iran. Patients were either admitted directly to the ICU or transferred from other wards, namely internal medicine, surgery, obstetrics, neurology, and cardiology wards. Post-operative patients requiring ventilation were admitted to the critical care unit, while patients with medical conditions necessitating ventilation were admitted to the ICU.

Overall, 71% of the hospitalized patients were male and 29% were female (*P ***≤ **0.5). The average age of the patients was 63 **± **0.8 years. The specimens were collected from the lung, blood, and urine of the patients hospitalized for 4 days in the ICU. A greater proportion of *Acinetobacter *spp. was isolated from lung aspirates (76%) than from urine samples (4%) from patients with urinary sepsis. The lung aspirates were homogenized with 5 mL 0.05 mM PIPS buffer and centrifuged at 8,000 rpm before being inoculated onto the medium.

The colonies on CLED agar were circular, smooth, convex, translucent, mucoid, and nonpigmented. They were gram-negative, encapsulated, non-spore-forming coccobacilli. The lactose utilization test for all isolates was negative. The organisms were nonmotile and nonhemolytic.

### Antibiotic susceptibility

The results of antibiotic susceptibility testing by the disk-diffusion test are shown in Table 1. Isolate numbers 1, 2, 4, 5, 10, and 15 were completely resistant to antibiotics routinely used in the ICU, while isolate no. 13 was the only one sensitive to most of the antibiotics. The emergence of resistance to imipenem, piperacillin, piperacillin + tazobactam, and ciprofloxacin is of particularly concern because these antibiotics are usually reserved for severely ill patients. The breakpoints obtained by disk-diffusion testing were further confirmed by determination of MICs, as shown in Table 2. For isolate numbers 1, 2, 5, 7, 9, 10, and 15, the MICs of imipenem, piperacillin, piperacillin + tazobactam, and ciprofloxacin were 240 µg/mL.

**Table 1:** Antibiotic susceptibility of *Acinetobacter *spp. isolated from the ICU of Afzalipoor Hospital.

Acinetobacter Isolates	CTX	CPM	CZ	CP	PIP	CAZ	IMP	Te	Gm	AK	PIT	C
**1**	R	R	R	R	R	R	R	R	R	R	R	R
**2**	R	R	R	R	R	R	R	R	R	S	R	R
**3**	R	R	R	S	R	S	S	R	I	I	R	R
**4**	R	R	R	R	R	R	R	R	R	I	R	R
**5**	R	R	R	S	R	R	R	R	R	I	R	R
**6**	R	R	R	R	R	R	S	R	I	S	R	R
**7**	R	R	R	R	R	R	R	R	I	R	R	I
**8**	R	R	R	S	R	R	S	S	R	R	R	R
**9**	R	R	R	R	I	R	R	R	R	I	R	R
**10**	R	R	R	R	R	R	R	R	R	R	R	R
**11**	R	R	R	S	R	I	S	R	I	S	R	R
**12**	R	R	R	R	R	R	R	I	S	S	R	R
**13**	S	R	R	S	I	S	S	R	S	S	S	I
**14**	R	R	R	I	R	R	R	R	R	S	R	I
**15**	R	R	R	R	R	R	R	R	R	I	R	R

Susceptibility was determined by the disk-diffusion breakpoint method.

CTX = cefotaxime, CPM = cefepime, CZ = cefazolin, CP = ciprofloxacin, PIP = piperacillin, PIT = piperacillin + tazobactam, CAZ = ceftazidime, IMP = imipenem, Te = tetracycline, Gm = gentamicin, AK = amikacin, C = chloramphenicol, R = resistant, I = intermediate, S = sensitive. Sterile Muller-Hinton agar was used for determination of antibiotic susceptibility and the inoculum concentration was 1.5 × 10^7^

**Table 2:** Minimum inhibitory concentrations (MICs) of different antibiotics for *Acinetobacter *spp. isolated from the ICU of Afzalipoor Hospital.

Acinetobacter isolates	MIC* (µg/mL)										
	**CPM**	**Gm**	**AK**	**Am**	**PIT**	**CAZ**	**C**	**PIP**	**Te**	**IMP**	**CP**
1	120	240	128	>240	>240	>240	60	>240	>240	240	>240
2	30	240	4	>240	>240	>240	>240	>240	>240	240	>240
3	10	10	4	240	30	240	>240	>240	>240	4	2
4	240	>240	16	>240	>240	>240	60	>240	>240	128	128
5	>240	>240	16	>240	240	>240	60	>240	>240	240	>240
6	30	5	4	240	120	240	120	128	>240	10	5
7	240	>240	32	>240	>240	>240	60	>240	>240	240	240
8	60	240	64	>240	5	>240	10	128	4	10	4
9	240	>240	16	240	>240	240	>240	16	>240	240	>240
10	240	>240	128	>240	>240	>240	120	>240	>240	240	>240
11	10	5	4	240	60	240	120	128	16	10	4
12	60	5	32	>240	>240	>240	120	>240	>240	128	>240
13	5	5	4	>240	30	5	10	16	>240	4	5
14	60	120	4	>240	60	>240	10	>240	>240	128	64
15	30	240	4	240	30	240	60	128	240	240	240

*MIC for each antibiotic was determined twice by the E-test on Mueller-Hinton agar, and CFU/mL was kept at 1.5 × 10^7^.

CPM = cefepime, Gm = gentamicin, AK = amikacin, Amp = ampicillin, PIT = piperacillin + tazobactam, CAZ = ceftazidime, C = chloramphenicol, PIP = piperacillin, Te = tetracycline, IMP = imipenem, CP = ciprofloxacin.

**Figure 1:**
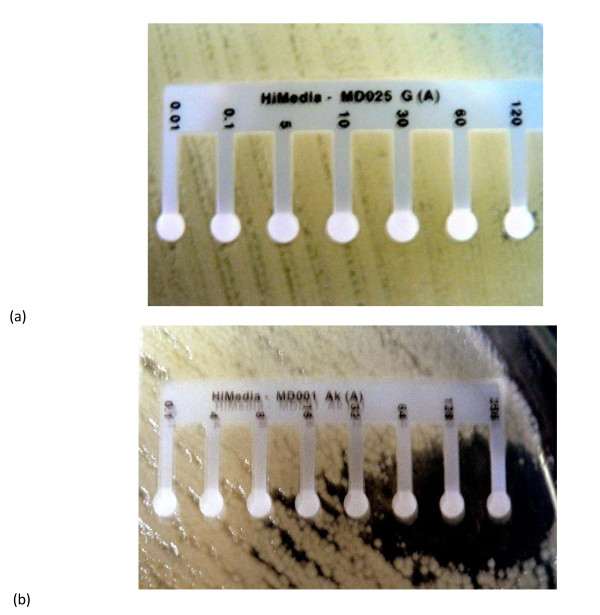
**MICs of gentamicin [Gm (a)] and amikacin [AK (b)] for *Acinetobacter *isolates as determined by the E-test**. The MIC was measured as lowest concentration of the antibiotic that inhibits the visible growth of the organism. Mueller-Hinton agar with initial CFU/mL 1.5 × 10^7 ^after 24 hours of incubation at 37°C was used as medium.

All of the *Acinetobacter *isolates were also highly resistant to third-generation cephalosporins (cefotaxime and ceftazidime), with MICs exceeding 240 µg/mL. For isolate number 13, the MICs of imipenem and amikacin were each 4 µg/mL, and the MICs of ceftazidime + cefotaxime and ciprofloxacin were each 5 µg/mL. Figure 1 (a and b) shows the MICs of gentamicin and amikacin for *Acinetobacter *isolates 1 and 12, respectively, as determined by the E-test. The imipenem-resistant isolates were multiresistant, but, most (46%) were sensitive to amikacin using the CLSI breakpoint of 4µg/mL. The important observation was the complete resistance of all isolates when tested with the amoxicillin + clavulanic acid combination disk (Table 2).

### ESBL production

The DDST method was used to detect the production of ESBL, and results were confirmed by the presence of a zone of inhibition around a disk containing a combination of the cephalosporin (ceftazidime) and clavulanic acid when compared with the zone around a disk containing the cephalosporin (ceftazidime) alone. Only three isolates (nos. 1, 10, and 15) were capable of producing ESBL. The remaining isolates exhibited a high degree of resistance to third-generation cephalosporins but did not produce ESBL.

### Substrate hydrolysis

The rates of substrate hydrolysis and the inhibitor profiles of β-lactamase produced by the *Acinetobacter *isolates from the ICU of Afzalipoor Hospital are shown in Table 3. The highest rate of ceftazidime and cefotaxime hydrolysis was observed in the presence of p-CMB (80.2 **± **0.02 µM/mL), while lowest rate was observed when NaCl (2.1 **± **0.01 µM/mL) was used as substrate (*P ***≤ **0.05). The rate of substrate hydrolysis decreased in the presence of sulbactam (Table 3). To eliminate the possibility of false-positive ESBL tests due to intrinsic susceptibility of β-lactamase inhibitors or the other mechanisms, the MIC of cefotaxime and ceftazidime for the ESBL-producing isolates was measured in the presence and absence of the β-lactamase inhibitor clavulanic acid. The MICs of ceftazidime and cefotaxime were decreased from > 240 µg/mL to 8 µg/mL and 4 µg/mL, respectively.

**Table 3:** Rates of substrate (β-lactam) hydrolysis and inhibitor profiles of *Acinetobacter *spp. isolated from the ICU of Afzalipoor Hospital.

Substrate	MIC (µg/mL)	β-lactamase inhibitor	Substrate hydrolysis (µmol)
CTX	>240	-	80**±**0.04
CTX	>240	P-CMB (50mM)	80.2**±**0.02
CTX	>240	NaCl (100mM)	2.1**±**0.01
CTX	>240	Sulbactam (200µg/ml)	10.2**±**0.03
CTX	>240	Clavulanic (200µg/ml)	7.5**±**0.02
CAZ	>240	-	80**±**0.04
CAZ	>240	P-CMB (50mM)	80.2**±**0.02
CAZ	>240	NaCl (100mM)	2.1**±**0.01
CAZ	>240	Sulbactam (200µg/ml)	30.2**±**0.03
CAZ	>240	Clavulanic (200µg/ml)	7.5**±**0.02

β-lactamase inhibitors were added 15 min before addition of the substrate.

*P *≤ 0.05 was considered significant of association. The above results represent the mean of two independent experiments. Means and standard deviations (SD) were calculated as required for numerical variables.

CTX = cefotaxime, CAZ = ceftazidime.

## Discussion

*Acinetobacter *spp. are resistant to the most commonly available antibiotics; hence, they are able to survive in the hospital environment under hostile conditions and also to colonize susceptible patients treated with broad-spectrum antibiotics. They are able to survive on various surfaces (both moist and dry) in the hospital environment [19,20]. An outbreak of *Acinetobacter *respiratory tract infection resulting from incomplete disinfection of ventilator equipment was reported by Cefai et al. [21]. In our investigation, specimens were collected from the lung, blood, and, in one case (isolate 12), urine of severely ill patients hospitalized in the ICU for 4 days. The average of age of the infected patients was 63 **± **0.8 years. The MICs of antibiotics routinely used in the ICU of our hospital indicated the emergence of resistance in some *Acinetobacter *isolates (numbers 1, 2, 5, 7, 9, 10, and 15) to almost all the antibiotics used, including imipenem, ciprofloxacin, and piperacillin + tazobactam. These findings complicate the therapy of infections caused by *Acinetobacter *spp. It has been suggested that the overwhelming use of antibiotics, particularly fluoroquinolones (e.g., ciprofloxacin) and carbapenems (e.g., imipenem), has resulted in the emergence of more resistant forms of colonizing strains [6]. ESBL was produced by three isolates, while the remaining isolates demonstrated no enzymatic activity. These results suggest that intrinsic resistance to antibiotics in non-ESBL-producing isolates may be due to mutation(s) in the genome involved in antibiotic susceptibility, resulting in high MIC values. The important observation was the insensitivity of the *Acinetobacter *isolates to amoxicillin + clavulanic acid, despite susceptibility to ceftazidime + clavulanic acid.

In a recent study, a DDST method that combined amoxicllin + clavulanic acid with cefepime were successfully detected the SHV-5 β-lactamase in a *Klebsiella pneumoniae *strain that produced a plasmid-borne AmpC enzyme [22]. In another report, the use of cefepime increased the sensitivity of the DDST with extended-spectrum cephalosporins for the detection of ESBLs in enterobacteria from 16% to 61% when the disks were applied at the standard distance of 30 mm from amoxicillin + clavulanic acid and from 71% to 90% when disks were applied at a shorter distance (20 mm) [23]. These results suggested that the inh ibition of the activities of the AmpC enzyme and efflux pumps might enhance the abilities of DDST to detect ESBLs in *Pseudomonas aeruginosa*.

In our study, the rate of hydrolysis of ceftazidime/cefotaxime was highest in the presence of p-CMB and lowest when NaCl and clavulanic acid (200 µg/ml) were used as substrate (*P ***≥**0.05). The results possibly explain the expanded zone of inhibition around the ceftazidime/cefotaxime + clavulanic acid disk and confirmed that the ESBL production among the isolates was not due to the intrinsic sensitivity of *Acinetobacter *isolates to β-lactamase inhibitors.

The results of antibiotic susceptibility testing of gram-negative bacilli strains isolated from the ICU of the Fundeni Clinical Institute, Bucharest, Romania [4], showed that 80% of 19 strains of *Acinetobacter *spp. isolated from nasal and pharyngeal exudates and bronchial secretions from immune-deficient patients were highly resistant to imipenem and were also resistant to the majority of the antibiotics tested.

Similarly, in one study on *Acinetobacter *susceptibility in Iran [24], it was found that the rates of sensitivity of the isolates to imipenem, piperacillin + tazobactam, and Amikacin were 50.7%, 50%, and 38.2%, respectively. However, it has recently become obvious that increased expression of chromosomal genes for efflux systems plays a major role in multidrug resistance [25]. Lagatolla et al. [26] reported that MICs of imipenem for the *bla*VIM-positive isolates were always **≥ **64 µg/mL (range 64-512 µg/mL). Most of the *bla*VIM-positive isolates (49 of 64 [76%]) exhibited a multidrug-resistant phenotype that included all of the drugs tested (imipenem, meropenem, ceftazidime, piperacillin, aztreonam, amikacin, gentamicin, tobramycin, and ciprofloxacin) [26]. In one outbreak in French hospital [27], twelve clonally related and multidrug-resistant *Acinetobacter baumannii *isolates were recovered during a 4-month period from 12 patients hospitalized at the Valenciennes Hospital. Seven clonally related *bla*_VEB-1 _positive *A*. *baumannii *strains were identified in the immediate environment of the hospitalized patients.

We found similar results in our study with isolate no. 1, which might be a carbapenemase-producing, *bla*VIM-positive isolate. Further research must be conducted to determine the mechanism of resistance to the above antibiotics.

## Conclusions

The results of this study showed that the majority of ICU isolates of *Acinetobacter *spp. were highly resistant to the antibiotics most commonly used in the ICU setting, including imipenem, ciprofloxacin, cefepime, and piperacillin + tazobactam, all of which are already used extensively in Iranian hospitals. Tests for ESBL production and substrate hydrolysis by resistant strains revealed the unique property of the ESBL (sensitivity to cefotaxime + clavulanic acid and to ceftazidime + clavulanic acid, and high rate of hydrolysis by p-CMB) produced by our isolates. These findings have important implications for physicians, microbiologists, and hospital administrators involved in the treatment of ICU patients in Iran.

## Competing interests

The authors declare that they have no competing interests.

## Authors' contributions

MRS and SA have made substantive intellectual contributions to this study. MHS helped in the laboratory preparation and setting. All authors read and approved the final manuscript.
